# A Novel Dual-Permanent-Magnet Mechanical Antenna for Pipeline Robot Localization and Communication

**DOI:** 10.3390/s23063228

**Published:** 2023-03-17

**Authors:** Yahao Dong, Jing Wu, Xinran Zhang, Tianyu Xie

**Affiliations:** School of Automation Science and Electrical Engineering, Beihang University, Beijing 100191, China

**Keywords:** mechanical antenna, ultra-low frequency, amplitude modulation, communication

## Abstract

The demand for pipeline inspection has promoted the development of pipeline robots and associated localization and communication technologies. Among these technologies, ultra-low-frequency (30–300 Hz) electromagnetic waves have a significant advantage because of their strong penetration, which can penetrate metal pipe walls. Traditional low-frequency transmitting systems are limited by the size and power consumption of the antennas. In this work, a new type of mechanical antenna based on dual permanent magnets was designed to solve the above problems. An innovative amplitude modulation scheme that involves changing the magnetization angle of dual permanent magnets is proposed. The ultra-low-frequency electromagnetic wave emitted by the mechanical antenna inside the pipeline can be easily received by the antenna outside to localize and communicate with the robots inside. The experimental results showed that when two N38M-type Nd–Fe–B permanent magnets with a volume of 3.93 cm^3^ each were used, the magnetic flux density reached 2.35 nT at 10 m in the air and the amplitude modulation performance was satisfactory. Additionally, the electromagnetic wave was effectively received at 3 m from the 20# steel pipeline, which preliminarily verified the feasibility of using the dual-permanent-magnet mechanical antenna to achieve localization of and communication with pipeline robots.

## 1. Introduction

Oil and gas pipelines underground and underwater need to be tested to ensure their safety, which necessitates the development of pipeline detection robot systems [1,2,3]. Achieving localization of and communication with cable-free pipeline robots is one of the key problems to be solved [4]. There are several localization methods, including the wheel odometer method, ray method, acoustic method, and electromagnetic wave method. The wheel odometer method can only provide off-line position information, and skidding between the wheels and pipe wall and wheel-locked situations result in accumulated position errors [5]. To improve its accuracy, some signal-processing methods including the prior-backtracking, data fusion, and point cloud registration methods have been proposed and achieved good results [6,7]. The ray method uses rays released by radioactive elements to achieve localization of robots. Although these rays can easily penetrate metal pipe walls, they are harmful to biological systems and the environment [8]. The acoustic method uses acoustic sensors to monitor the robot’s position, but it is susceptible to environmental noise and requires high sensitivity of the acoustic sensors. Moreover, when a robot stops running due to unexpected factors, it cannot be located [9,10]. The electromagnetic wave method uses magnetic sensors to receive electromagnetic wave signals emitted by the pipeline robots for localization with little external interference [11,12,13]. However, metal pipelines shield high-frequency electromagnetic waves. In contrast, ultra-low-frequency electromagnetic waves have low attenuation and can penetrate metal pipelines, making this the preferred frequency band for localization of and communication with pipeline robots.

Conventional low-frequency antennas generate electromagnetic waves through an energized coil [14,15,16], and the power loss and volume of equipment are inevitably large. Permanent-magnet-type mechanical antennas have recently received wide attention in the field of underwater and underground communications. They can produce a strong magnetic field by using a small permanent magnet of high magnetic energy density [17], which provides a new method for the localization of and communication with pipeline robots [18].

According to their structure, permanent-magnet-type mechanical antennas can be mainly classified into three types. (a) Modulation is realized by changing the rotational speed of the permanent magnets [19,20,21,22]. For example, the frequency of the signal can be modulated by adjusting the speed of a three-phase induction motor. This type is easy to implement, but the power consumption is large and the bandwidth is narrow due to the inertia of the magnets. (b) The permanent magnet rotation speed is maintained as constant and a modulator is added to achieve the signal modulation. For example, when placing a cylinder with a coil wrapped around a spherical permanent magnet rotating at a constant speed, the amplitude of the magnetic field can be modulated by controlling the coil current [23,24]. The phase of the magnetic field can be modulated by changing the position of two pairs of orthogonal bow-tie-shaped shielding layers [25,26]. Such schemes do not require the speed of the motor to be changed, reducing the power consumption. However, the additional modulator increases the complexity of the system. (c) The permanent magnet is maintained at rest and signal modulation is realized through a special modulator. For example, the frequency of the magnetic field can be modulated by varying the rotational speed of a louvered rotary shutter structure made from soft magnetic materials which periodically shields the magnetic field generated by the magnets [27]. This type reduces the requirement for mechanical strength of the system, as the permanent magnets are stationary. However, it has high demands regarding the structure and performance of the modulator.

Practical applications of the permanent-magnet mechanical antenna require the design of a suitable signal-modulation scheme considering the size, power consumption, and communication bandwidth of the antenna. The common modulation schemes for mechanical antennas are frequency modulation (FM), amplitude modulation (AM), and phase modulation (PM). Among them, AM can provide sufficient bandwidth and its implementation structure is not complex, but it is sensitive to noise [26]. In this work, a new AM scheme is proposed using a dual-permanent-magnet mechanical antenna with an innovative modulator. It has a large modulation depth which reduces the sensitivity to noise and is more suitable for the localization of and communication with pipeline robots. Its comparison with the existing modulation schemes is shown in Table 1.

The rest of this paper is organized as follows. Section 2 describes the structure and working principles of the new mechanical antenna. Section 3 presents its prototype and describes the experimental verification of its effectiveness in both air and pipelines. Finally, Section 4 provides the conclusions and discussion.

## 2. Theoretical Analysis of the Novel Mechanical Antenna

### 2.1. Theoretical Fundamentals

**Figure 1:**
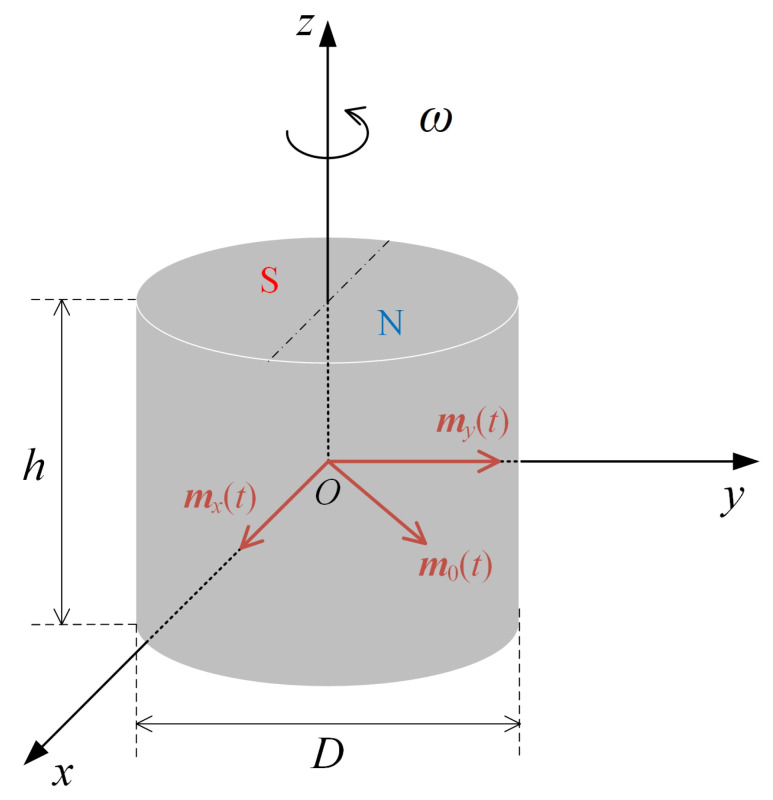
Equivalent model of a single rotating permanent magnet.

As shown in Figure 1, a cylinder-shaped permanent magnet with uniform magnetization along the radial direction is placed in infinite space. Its diameter and height are *D* and *h*, respectively, and its magnetic moment *m*_0_ = *M*_0_*V* where *M*_0_ is the magnetization intensity and *V* is the volume. A single stationary magnet can be equivalent to a magnetic dipole with the moment *m*_0_ when *r* >> max(*D*,*h*). If it rotates counterclockwise at a uniform angular velocity *ω*, the magnet can be equivalent to two time-varying dipoles orthogonal in both time and space, and this moment ***m***_0_(*t*) = *m_x_*(*t*)***e****_x_*+ *m_y_*(*t*)***e****_y_* and $\dot{m}$*_x_* = *j*$\dot{m}$*_y_*.

In a spherical coordinate system, the magnetic flux density generated by the dipole *m_y_* can be expressed as follows:

(1) $$\begin{array}{ll} {\dot{B}}_{m_{y}}(r,\theta ,\varphi ,{\dot{m}}_{y}) & =\frac{\mu_{0}{\dot{m}}_{y}k^{3}}{2\pi }e^{-jkr}\left[\frac{j}{{\left(kr\right)}^{2}}+\frac{1}{{\left(kr\right)}^{3}}\right]\sin\theta \sin\varphi e_{r}\\ & -\frac{\mu_{0}{\dot{m}}_{y}k^{3}}{4\pi }e^{-jkr}\left[-\frac{1}{kr}+\frac{j}{{\left(kr\right)}^{2}}+\frac{1}{{\left(kr\right)}^{3}}\right]\cos\theta \sin\varphi e_{\theta }\\ & -\frac{\mu_{0}{\dot{m}}_{y}k^{3}}{4\pi }e^{-jkr}\left[-\frac{1}{kr}+\frac{j}{{\left(kr\right)}^{2}}+\frac{1}{{\left(kr\right)}^{3}}\right]\cos\varphi e_{\varphi } \end{array}$$

where *k* is the propagation constant and *k* = *ω*$\sqrt{\varepsilon_{0}\mu_{0}}$ in which *μ*_0_ and *ε*_0_ are the permeability and dielectric constant of the vacuum, respectively. The magnetic flux density generated by the dipole *m_x_* can be expressed as follows:

(2) $${\dot{B}}_{m_{x}}(r,\theta ,\varphi ,{\dot{m}}_{y})={\dot{B}}_{m_{y}}(r,\theta ,\varphi +\frac{\pi }{2},j{\dot{m}}_{y})$$

Therefore, the magnetic flux density generated by a single rotating permanent magnet is as follows:

(3) $${\dot{B}}_{s}(r,\theta ,\varphi )={\dot{B}}_{m_{x}}(r,\theta ,\varphi )+{\dot{B}}_{m_{y}}(r,\theta ,\varphi )$$

**Figure 2:**
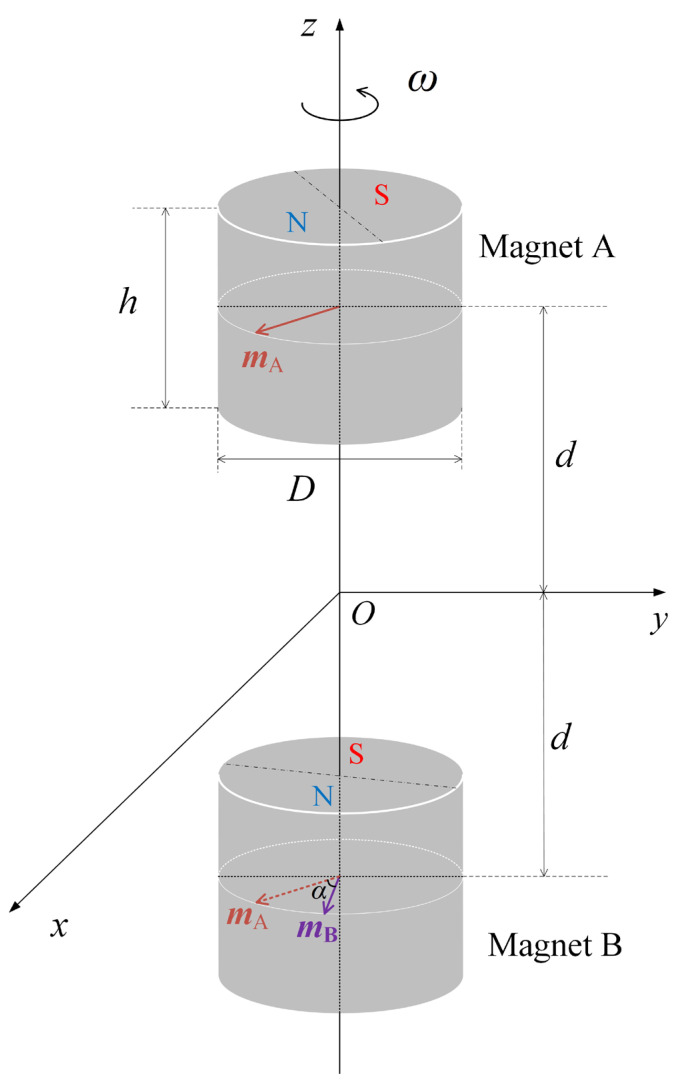
Dual-permanent-magnet mechanical antenna model.

Based on the magnetic field distribution of a single rotating permanent magnet, the dual-permanent-magnet mechanical antenna model is presented, as shown in Figure 2. In this model, the permanent magnets A and B rotate uniformly around the *z* axis with the same angular velocity *ω*, and their centers are both at a distance *d* from the coordinate origin *O*. The dimensions of the permanent magnets are same as those in Figure 1, and the angle between the magnetic moments is *α*. Assume that the magnetic flux densities generated by *A* and *B* are expressed by ${\dot{B}}_{A}$ and ${\dot{B}}_{B}$, and then the magnetic flux density generated by the model shown in Figure 2 is as follows according to Equation (3):

(4) $$\begin{array}{ll} {\dot{B}}_{d}(x,y,z) & ={\dot{B}}_{A}+e^{j\alpha }{\dot{B}}_{B}\\ & ={\dot{B}}_{s}(x,y,z-d)+e^{j\alpha }{\dot{B}}_{s}(x,y,z+d) \end{array}$$

In particular, when *r* >> max(*D*,*h*,*d*), the impact of *d* is ignored and *d* ≈ 0; thus,

(5) $${\dot{B}}_{d}(x,y,z)=(1+e^{j\alpha }){\dot{B}}_{s}(x,y,z).$$

**Figure 3:**
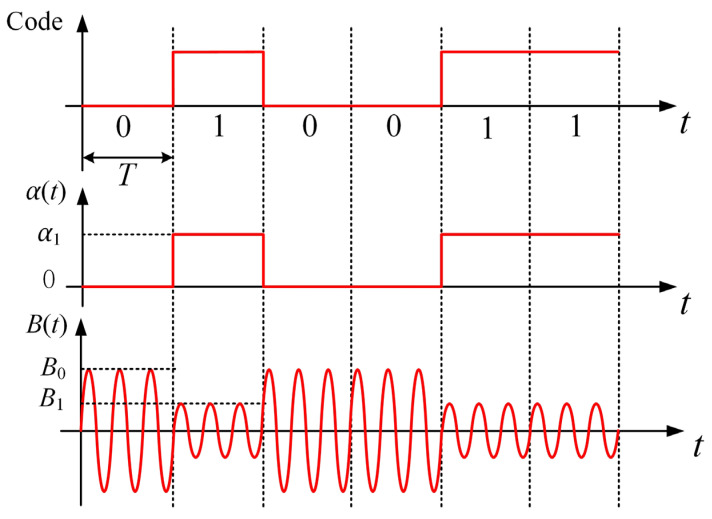
Waveforms of the amplitude modulation signal in an ideal case.

From Equation (5), the amplitude of ${\dot{B}}_{d}$ changes when *α* changes and amplitude modulation is achieved. Suppose that a message comprising code elements 0 and 1 must be sent. When element 0 is sent, let *α* = 0 and $B_{0}=\left|2{\dot{B}}_{s}\right|$. When element 1 is sent, let *α* = *α*_1_, and $B_{1}=\left|\left(1+e^{j\alpha_{1}}){\dot{B}}_{s}(x,y,z\right)\right|$. Figure 3 shows the waveforms of the ideal amplitude modulation signal in the time domain. If the difference between *B*_0_ and *B*_1_ is not significant and the ambient background magnetic field noise is large at the receiver, the quality of the amplitude modulation signal will become poor, which increases the bit error rate during demodulation. Here the modulation depth *T_m_* is defined as in Equation (6). According to Equation (5), $T_{m}=1-\cos(\alpha_{1}/2)$. *T_m_* can reach the value of 1 when *α* = 180°. The larger the *α*_1_, the larger the *T_m_*, and the stronger the antinoise performance of the system and the more reliable the communication.

(6) $$T_{m}=\left|\frac{B_{1}-B_{0}}{B_{0}}\right|=\left|\frac{\left|{\dot{B}}_{s}(x,y,z-d)+e^{j\alpha_{1}}{\dot{B}}_{s}(x,y,z+d)\right|-\left|{\dot{B}}_{s}(x,y,z-d)+{\dot{B}}_{s}(x,y,z+d)\right|}{{\dot{B}}_{s}(x,y,z-d)+{\dot{B}}_{s}(x,y,z+d)}\right|$$

### 2.2. Effectiveness of the Analytical Formula

**Figure 4:**
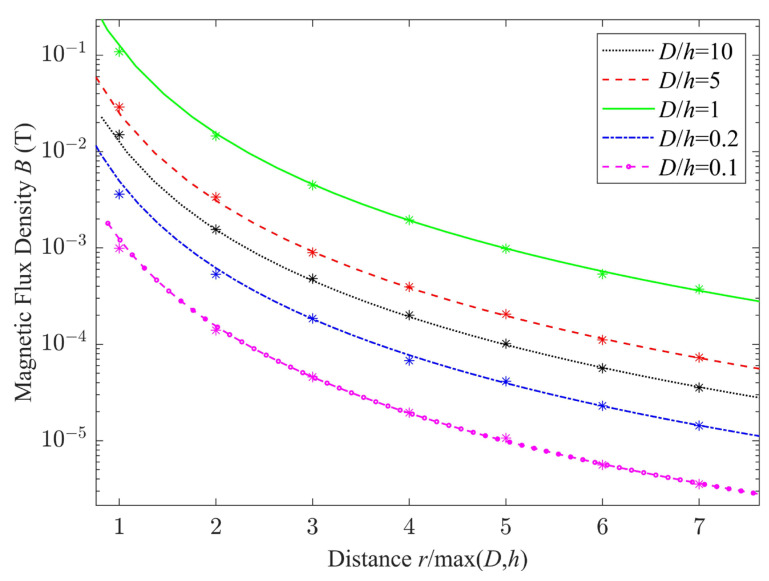
Comparison of the results of the analytical solution in Equation (4) with those obtained via the finite-element calculation for different ratios of cross-sectional diameter of the permanent magnet to its height (*D*/*h*). Asterisks indicate simulation results and lines indicate analytical results.

The derivation of Equation (4) requires that a single rotating permanent magnet be equivalent to two time-varying magnetic dipoles. The effectiveness of this equivalence is verified by maintaining the volume *V* of the cylindrical permanent magnet as a constant and changing the outer dimension, i.e., the ratio of cross-sectional diameter to height *D*/*h*. The magnetic field distribution obtained using Equation (4) is compared with that obtained using finite-element calculations. Assume a radially magnetized permanent magnet is N38M-type and its volume *V* = 3.93 cm^3^, and the rotation frequency *f* = 30 Hz. For different values of (*D*/*h*), the magnetic field distribution along the *y* axis direction is shown in Figure 4, in which the errors are considerable when the spatial position *r* is close to the permanent magnets. However, when *r*/max(*D*,*h*) ≥ 4, the errors between two methods are approximately less than 5%, in which case the magnetic field calculated using Equation (4) is more accurate and the influence of the magnets’ dimensions can be ignored.

## 3. Development of the Novel Mechanical Antenna

### 3.1. Dual-Permanent-Magnet Mechanical Antenna

**Figure 5:**
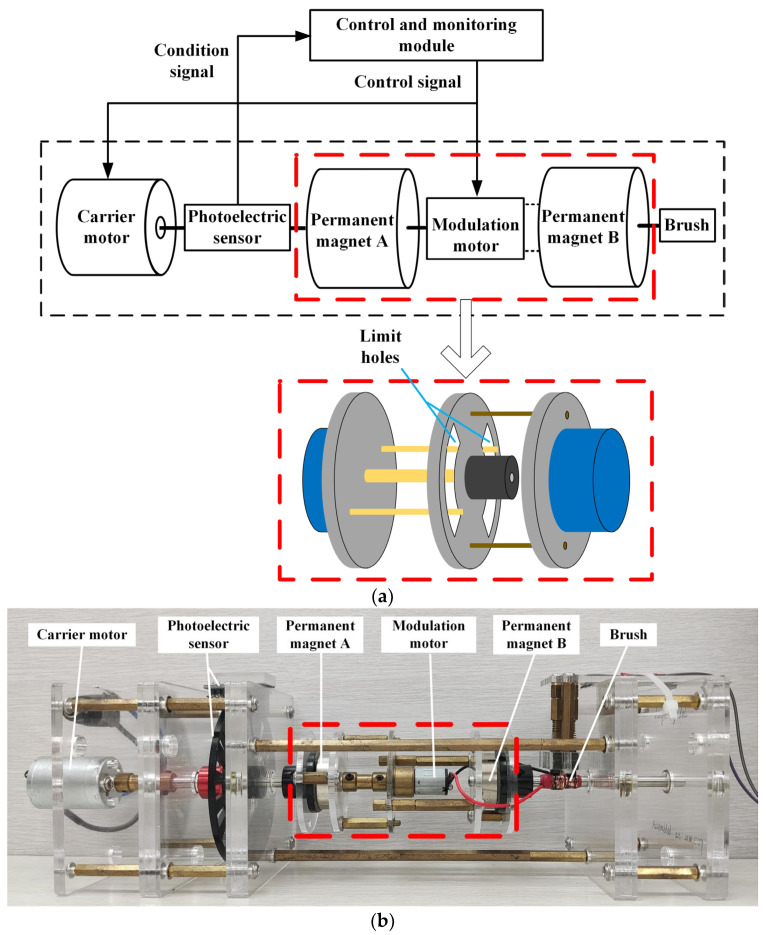
Dual-permanent-magnet mechanical antenna: (**a**) principle of the structure; (**b**) physical prototype.

The structure of the dual-permanent-magnet mechanical antenna is shown in Figure 5a and the physical prototype of the antenna is shown in Figure 5b. It consists of a carrier module, a modulation module, and a control and monitoring module. The carrier module comprises a carrier motor, a rotating shaft, and permanent magnets A and B, in which the carrier motor drives A and B through the rotating shaft to rotate at a constant speed so that the magnetic field is radiated outward steadily. In Figure 5b, the carrier motor is a 12 V RK-370CA permanent magnet DC motor with a maximum speed of 5400 rpm, and both A and B are N38M-type radially magnetized permanent magnets with a remanence of 1.25 T, *D* = 25 mm, *h* = 8 mm, and *d* = 32.5 mm. The modulation module comprises a modulation motor, limiters, and brushes. The modulation motor is a 6 V geared motor rated at 300 rpm which is used to adjust the angle *α* of magnetic moments between A and B, and the limiters can limit the value of *α*. The modulation motor rotates along with the rotating shaft of the carrier motor, and the brushes are installed to prevent the wires at the outlet of the modulation motor from becoming entangled during the rotation process. The control and monitoring module comprises an Arduino UNO microcontroller, an AQMH2407ND DC motor driver, and a photoelectric sensor, which are used to control the rotation of the carrier and modulation motors and collect their status information through the photoelectric sensor. The power grid working frequency of 50 Hz should be avoided as the operating frequency of the antenna. The smaller the operating frequency, the deeper the wave will penetrate underground and underwater.

### 3.2. Experiment in Air

**Figure 6:**
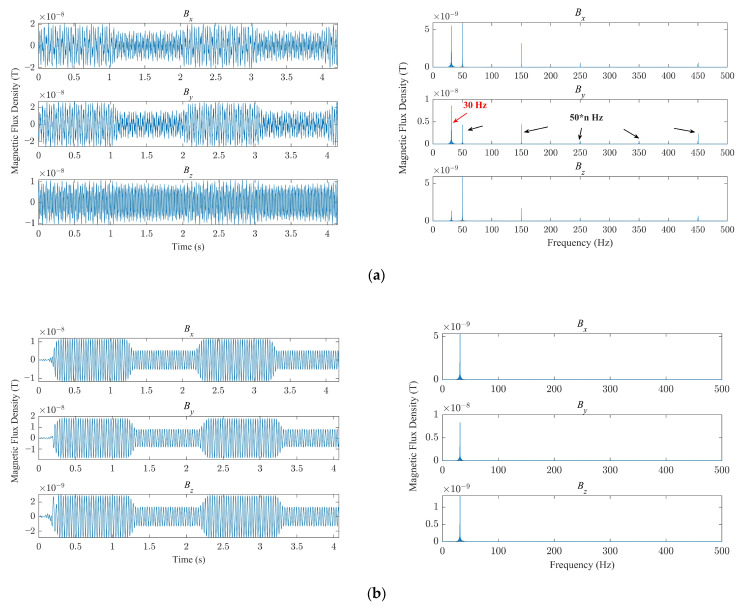
Three-dimensional magnetic flux density and its spectral characteristics: (**a**) before filtering; (**b**) after filtering.

**Figure 7:**
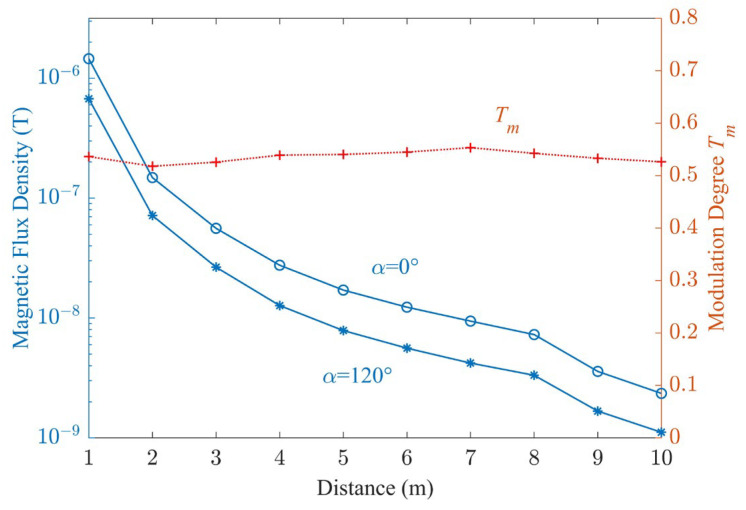
The distribution of magnetic flux density *B* and modulation depth *T_m_*.

The operating frequency of the antenna was maintained at 30 Hz. By switching *α* between 0° and 120°, the antenna transmitted codes 0 and 1 at a rate of 1 bit/s. Figure 6a shows the waveforms of the magnetic flux densities *B_x_*, *B_y_*, and *B_z_* measured at *y* = 5 m along the positive *y* axis using a three-dimensional symmetrical induction coil [28]. In addition to the spectral component at 30 Hz, the components at 50 Hz and its harmonic frequencies were also measured. The waveforms after filtering these components are shown in Figure 6b. The distribution of magnetic flux density *B* and the modulation depth *T_m_* along the positive *y* axis are shown in Figure 7 when *α* = 0 and *α* = 120°. It can be seen the magnetic field reached 2.35 nT at *y* = 10 m when *α* = 0 and the values of *T_m_* ranged from 50% to 60%.

### 3.3. Simulation and Experiments in Pipelines

**Figure 8:**
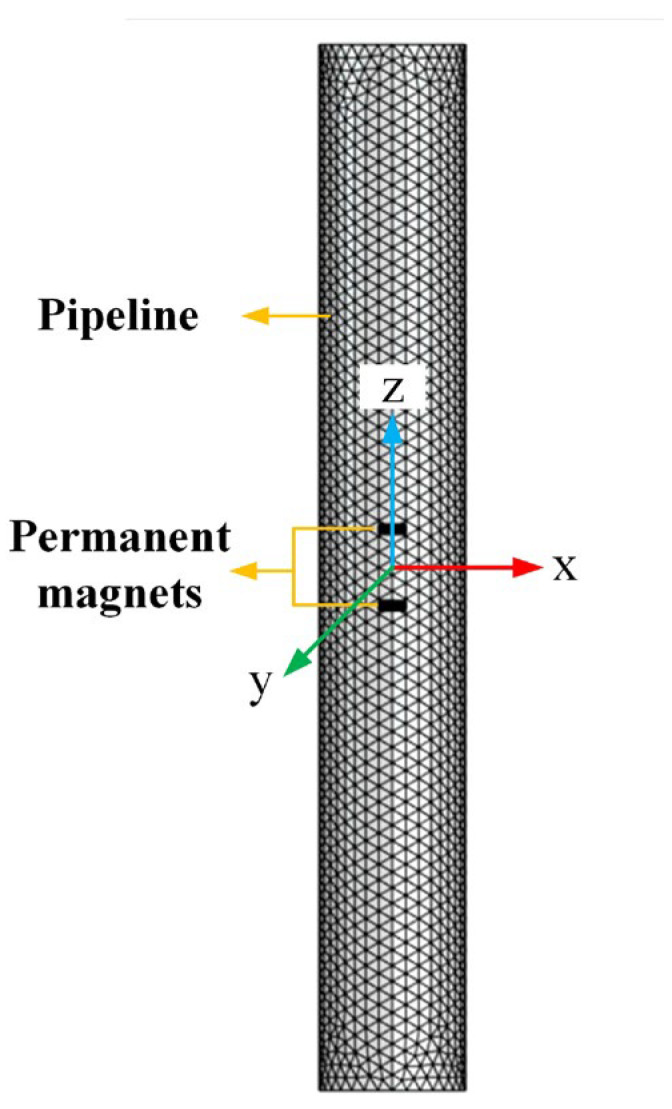
The simulation model.

Due to the shielding effect of the metal pipe wall, when the antenna is placed inside a pipeline, the magnetic field signal will be weakened. To verify the effectiveness of the antenna in a pipeline, finite-element simulations were conducted. The simulation model is shown in Figure 8, wherein the mechanical antenna was placed at the center of the pipeline. The pipeline’s outer radius *r*_p_ = 0.07 m, length *l*_p_ = 1 m, and thickness *h*_p_ = 0.005 m. The material of the pipeline was set to be air (no pipeline), aluminum, or iron. The aluminum’s conductivity *σ*_Al_ = 3.77 × 10^7^ S/m. The iron’s relative permeability *µ*_Fe_ = 4000 and conductivity *σ*_Fe_ = 1.12 × 10^7^ S/m. The magnets had a diameter *D* = 0.025 m, height *h* = 0.008 m, distance 2*d* = 0.065 m, and rotation frequency *f* = 30 Hz.

**Figure 9:**
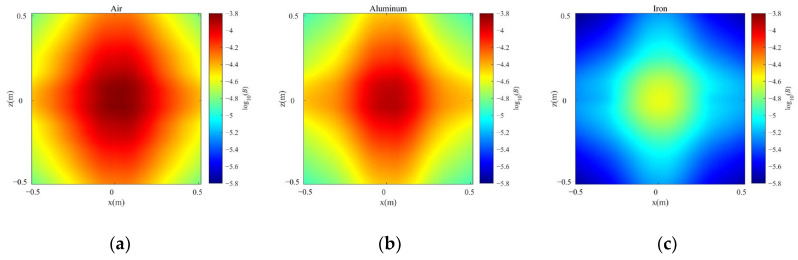
The distributions of magnetic flux density: (**a**) air; (**b**) aluminum; (**c**) iron.

The distribution of magnetic flux density *B* is shown in Figure 9 when *y* = 0.2 m. It can be seen that when the antenna was placed in the pipeline, *B* was about 10^−5^ T and it was attenuated due to the shielding effect of the pipe wall. The shielding effect of the iron pipeline with high permeability was more obvious than that of the aluminum pipeline.

**Figure 10:**
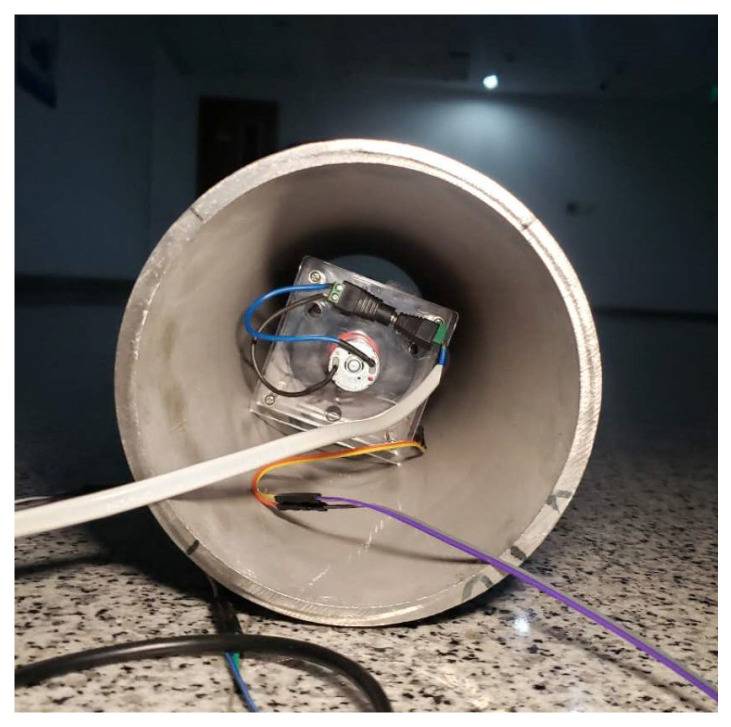
The mechanical antenna placed in an actual 20# steel pipeline.

Pipelines made of 20# steel are mainly used for boilers and heat exchangers to transport fluids, which are widely used in petrochemical, power stations, and large-scale equipment. In the experiment, the antenna was placed in a practical 20# steel pipeline with an outer radius of 180 mm, length of 1200 mm, thickness of 8 mm, and conductivity of about 4.22 MS/m, as shown in Figure 10. The measured results are shown in Table 2. It can be seen that when a 20# steel pipeline was used, the magnetic flux density was effectively received at 3 m from the carbon steel pipeline with a receiving antenna able to measure a magnetic flux density of as low as 0.15 pT. *B_x_*, *B_y_*, and *B_z_* all had high attenuation which was consistent with the simulation results. When *α* was changed, the amplitudes of *B_x_*, *B_y_*, and *B_z_* changed significantly, indicating a good modulation.

## 4. Conclusions and Discussion

For pipeline robot positioning, compared with the wheel odometer, ray, and acoustic wave methods, the proposed method utilizes ultra-low-frequency electromagnetic waves with low attenuation, long transmission distance, and strong penetrability generated by the new small mechanical antenna made of permanent magnets. In this method, the amplitude modulation of the magnetic flux density is realized by changing the angle of the magnetic moments between the two permanent magnets. The effects of the mechanical antenna size on the magnetic field signal were studied using analytical theory and finite-element simulations. When *r*/max(*D*,*h*) ≥ 4, the influence of the magnets’ outer dimensions can be ignored. The experimental results showed that using the developed antenna, the quality of magnetic signal was satisfactory for communication at 10 m in the air and 3 m from a 20# steel pipeline, which preliminarily verified the feasibility of the antenna for localizing and communicating with pipeline robots.

The main factors affecting the communication quality of the proposed antenna can be summarized as follows:

a. The magnetization *M*_0_ and volume *V* of the permanent magnet. According to (3) and (4), the amplitude of the magnetic flux density increases linearly with *M*_0_ and *V*. Thus, *M*_0_ and *V* affect only the strength of the magnetic field and do not affect the modulation performance. Increasing *M*_0_ and *V* can improve the signal propagation distance. However, if *V* is substantially large, the overall volume and rotational inertia of the antenna will increase. Therefore, for a fixed propagation distance, a permanent magnetic material with a larger *M*_0_ can be selected without increasing the antenna volume.
b. The spacing *d* between the permanent magnets affects the near-field distribution and modulation performance of the antenna. When *r* >> max(*D*,*h*), the effect of *d* is negligible.
c. The rotational speeds *ω* of the carrier motor and *ω*_1_ of the modulation motor. Due to the limits of power consumption and long communication distance in conductive media such as underground or underwater, *ω* cannot be too large. However, the symbol transmission rate *v* (bit/s) depends on both *ω* and *ω*_1_. Assume the time required for the change of *α*: $\alpha =0\to \alpha_{1}$ or $\alpha =\alpha_{1}\to 0$ is *t*_1_, which is the switch time of the modulation motor from start to stop, and, to improve the demodulation accuracy at the receiver, the conditions $v<<1/t_{1}$ and v≤ω/π should be satisfied.

The new dual-permanent-magnet mechanical antenna has many application prospects in the field of communication through conductive media and finite space, such as in pipelines, underwater, undersea, and underground. However, there is still much work to be done before it can be applied. For the positioning of and communication with pipeline robots, its size needs to be further reduced, which can be implemented by using permanent magnets of higher magnetic energy density and optimizing the antenna structure. In addition, the current amount of data for validation is not very large. The antenna should be placed in industrial pipelines with different electromagnetic characteristics to test its ability to function despite electromagnetic interference from low-frequency atmospheric noise and noise from human sources, or the low-frequency magnetic field transmitted by the robot itself. Moreover, we also need to study the high-efficiency receiving antenna and the high-accuracy positioning algorithm based on the received signals. In the future, this method will become more practical.

## Figures and Tables

**Table 1:** Comparison of the antenna in this work with previously published works.

Modulation Type	Literature	Implementation	Characteristics
FM	[19,20,21,22]	Change drive motor’s speed	Simple structure High power consumption Low bandwidth
PM	[25,26]	Needs a modulator	Complicated structure Insensitive to noise Complex in demodulation
AM	[23,24]	Needs a modulator	Normal in structure Sensitive to noise
	This work	Needs a modulator	Normal in structure Lower sensitivity to noise

**Table 2:** Measured results.

Condition	*α* (°)	Distance (m)	*B_x_* (nT)	*B_y_* (nT)	*B_z_* (nT)
Without pipeline	0	3	40.57	38.35	4.43
With pipeline	0	3	6.59	5.40	0.67
Without pipeline	120	3	19.17	18.16	2.83
With pipeline	120	3	2.88	2.51	0.46

## Data Availability

Not applicable.
